# *Vepris macrophylla* (Baker) I. Verd Essential Oil: An Antifungal Agent against Phytopathogenic Fungi

**DOI:** 10.3390/ijms21082776

**Published:** 2020-04-17

**Authors:** Laura Giamperi, Anahi Elena Ada Bucchini, Donata Ricci, Bruno Tirillini, Marcello Nicoletti, Rianasoambolanoro Rakotosaona, Filippo Maggi

**Affiliations:** 1 Department of Biomolecular Sciences, Center of Research “Botanic Garden”, University of Urbino, 61029 Urbino, Italy; donata.ricci@uniurb.it (D.R.); bruno.tirillini@uniurb.it (B.T.); 2 Department of Environmental Biology, “La Sapienza” University of Rome, 00185 Rome, Italy; marcello.nicoletti@uniroma1.it; 3 Institut Malgache de Recherches Appliquées, Association-Fondation Rakoto Ratsimamanga, Avarabohitra Itaosy, Antananarivo 3833, Madagascar; rravalison@gmail.com; 4 School of Pharmacy, University of Camerino, 62032 Camerino, Italy; filippo.maggi@unicam.it

**Keywords:** *Vepris macrophylla*, essential oil, phytopathogenic fungi, mycotoxins, oxylipins, antifungal activity, inhibition of lipoxygenase

## Abstract

Rutaceae are widely used in ethnomedicine to treat infectious diseases in humans and plants. In this study, the antifungal activity of the *Vepris macrophylla* leaf essential oil (VEO) and its main components, citral and citronellol, was evaluated against six phytopathogenic fungi. In addition, the possible action of VEO on the synthesis of mycotoxins was evaluated as well. To determine the antifungal activity of VEO we used the agar dilution method and VEO showed inhibitory activity against all the tested fungi. In particular, VEO resulted to be fungicidal against *Phytophthora cryptogea* and *Fusarium avenaceum*. For all other fungi VEO exhibited fungistatic activity and the weakest effect was observed on *Alternaria solani*. Citral was very effective against *P. cryptogea*, *F. avenaceum*, *F. poae* and *F. graminearum*. On the other hand, citronellol showed good activity towards *P. cryptogea* and *F. avenaceum* and weaker activity towards *F. poae* and *F. graminearum*. It can be concluded that VEO can be considered a promising antifungal agent, especially against *P. cryptogea* and *F. avenaceum,* suggesting a possible use in the formulation of new selective and natural fungicides.

## 1. Introduction

Essential oils (EOs) are aromatic oily liquids hydrodistilled from several plant families which are basically made up of volatile secondary metabolites and play a vital role in the protection of plants against various biotic factors [1,2]. EOs and their active components are gaining attention in the pharmaceutical and perfume industry due to their herbal nature, versatile uses and wide acceptance [3,4,5]. Usually EOs are considered non-phytotoxic and highly active against various microbes [6]. Increasing bacterial resistance boosted scientific research on the antibacterial efficacy of EOs and plant derived compounds, demonstrating their use as food preservatives and their importance in the control of infectious diseases in humans and plants. Spoilage and poisoning of foods by fungi is a major problem, especially in developing countries. *Penicillium*, *Aspergillus* and *Fusarium* are the most important fungi causing spoilage of foodstuffs [7]. Fungi are also responsible for off-flavour formation and production of allergenic compounds and mycotoxins, which lead to qualitative losses [7]. A number of important mycotoxins have been isolated from the genus *Fusarium*. Thus, adequate control measures to prevent spoilage of grains and foodstuffs are essential to avoid contamination and minimize public health hazards.

In areas that have a great ecological wealth but are burdened by intense economic poverty, such as Madagascar, the population, due to the inaccessibility and the prohibitive costs of Western medicine, uses traditional medicine to meet most healthcare needs. The knowledge of native medicinal plants, and the local production of pharmaceutical products based on plant derivatives offer a valid alternative to Western medicine. [8,9,10]. The rich vegetation of Madagascar also includes the Rutaceae family which encompasses about 1600 species, characterized by the presence of oil glands producing aromatic ethereal oils. The genus *Citrus* and the genus *Vepris* belong to this family [8,9,10]. The genus *Vepris* includes around 80 species mainly occurring in the tropical areas of Africa, Arabia and India. Among them, 28 are endemic to Madagascar such as *Vepris macrophylla* (Baker) I. Verd (syn. *Toddalia macrophylla* Baker). Concerning the EOs of the genus *Vepris*, five species growing in Madagascar have been investigated so far [11]. The chemical composition of *V. madagascarica* EO is characterized by α-pinene, *p*-cymene, eugenol, methyl eugenol and estragole as the main constituents [12]. In another study, (*E*)-anethole (78.2–84.6%) was found as the main volatile component in *V. madagascarica* leaf and trunk bark [13]. The *Vepris elliotii* EO composition was characterized by terpinolene (49.7%) and (*E*)-anethole (23.5%) [14]. The main components of *Vepris leandriana* EO were citronellol (33.2–33.6%), geranial (27.0–33.0%) and neral (19.5–21.8%) [15].

The EO of *V. macrophylla* (VEO) was previously analyzed revealing geranial (33.2%), neral (23.1%) and citronellol (14.5%) as the major constituents [16]. Citral is the major component of lemongrass EO which was extracted from its leaves, present at levels of, approximately, 65–85%. As a natural acyclic monoterpene, citral was found in a wide variety of plants [17] including *V. macrophylla*. A number of dietary monoterpenes were shown to act effectively in chemoprevention and chemotherapy of different tumors in animal models, at the cellular level, and in human clinical trials [18]. Furthermore, unsaturated terpenes are capable of trapping activated oxygen species in vivo to give intermediate epoxides which can alkylate DNAs, proteins, and other biomolecules [18]. Due to the limitation of current antifungal drugs, their limited spectrum and the expensive treatment, new drugs and alternative therapies are necessary, including those derived from natural product compounds. Taking into account the therapeutic importance of monoterpene compounds, it is relevant to examine the citral as a novel treatment of fungal diseases in humans, animals and in plants.

From a botanical point of view, *V. macrophylla*, the subject of the present study, is an evergreen tree, occurring in subhumid forests, from sea level up to 600 m of altitude. In the traditional medicine the infusion from leaves and root are used as euphoristic, astringent, adaptogenic and antidepressive. In addition, the fruits are used as antiseptic [19,20].

Studies on the isolation of some acrylic alkaloids from the leaves of this plant, as well as on the composition and bioactivity of its EO are known in literature, whereas no reports on the antifungal activity against plant pathogens have been carried out.

Thus, the aim of this work, in continuation of our research on the chemical and antifungal activity of different EOs, was to test the inhibitory effects of VEO and its main compounds citral and citronellol against different phytopathogenic fungal strains such as four strains of *Fusarium* (*F. avenaceum, F. poae, F. graminearum, F. semitectum*), one strain of *Alternaria* (*A. solani*) and one strain of *Phytophthora* (*P. cryptogea*).

## 2. Results and Discussion

### 2.1. Chemical Analysis

The chemical composition of VEO showed a predominance of oxygenated monoterpenes (80.3%) such as citral (56.3%), an isomeric mixture of geranial (33.2%) and neral (23.1%), and citronellol (14.5%), and a lower amount of monoterpene hydrocarbons (10.6%) such as myrcene (8.3%) [16,20]. According to the chemical profile, VEO belongs to the ‘citral chemotype’ that has been already assigned to other *Vepris* EOs [16].

### 2.2. Antifungal Activity

From the literature, it is widely known that citral (3,7-dimethyl-2,6-octadienal) is the name given to a natural mixture of two isomeric acyclic monoterpene aldehydes, i.e., geranial (*trans*-citral, citral A) and neral (*cis*-citral, citral B), and that it is responsible for many biological activities [18]. In particular, its effectiveness as an antifungal agent against human and plant pathogens has been already demonstrated [18]. For this reason, in a program aimed at discovering natural fungicides as alternative to conventional synthetic agrochemicals, VEO was evaluated for antifungal activity using the agar dilution method against phytopathogenic fungi damaging crops of economic importance. Four strains of *Fusarium* (*F. avenaceum*, *F. poae*, *F. graminearum*, *F. semitectum*), one strain of *Alternaria* (*A. solani*) and one strain of *Phytophthora* (*P. cryptogea*), were utilized for the purpose. Results are shown in Table 1 and Table 2, which indicated that all concentrations tested of VEO inhibited the fungal growth.

In the agar dilution test VEO showed an inhibitory effect at 100, 200, 400 and 800 µg/mL against all tested fungi. *P. cryptogea* and *F. avenaceum* were found to be the most sensitive pathogens, with MIC values corresponding to 130.40 µg/mL (Table 2). In all tested fungi, the inhibitory effect of VEO was detected at 100 µg/mL and, on the first day of culture, ranging between 20 and 45% of inhibition (Table 1). At 800 µg/mL (major concentration tested) and on the third day of incubation VEO showed, against all fungi, an inhibition rate of over 50% and more precisely: 53.8% for *A. solani,* 60% for *F. poae*, 67% for *F. semitectum*, 71% for *F. graminearum*, 82.3% for *F. avenaceum* and 100% for *P. cryptogea*. Only against these last two fungi VEO at 390.30 µg/mL (MFC) showed fungicidal activity (Table 2). The weakest activity was observed against *A. solani* and *F. semitectum* which showed some resistance. In fact, at the highest concentration tested (800 µg/mL) and on the sixth day of incubation, the VEO effect on these two fungi was the lowest, with an inhibition rate of 73 and 78%, respectively. In this context we found interesting to evaluate the antifungal and fungicidal activity exerted by citral and citronellol. These results were compared with the VEO effect against *P. cryptogea*, *F. avenaceum*, *F. poae* and *F. graminearum.*

This is the first time that citral and citronellol were assayed against *F. avenaceum*, *P. cryptogea*., *F. poae* and *F. graminearum* (Table 2) because they were the most sensitive fungi. In this context citral was very effective against all these four fungi, while citronellol showed good activity towards *P. cryptogea* and *F. avenaceum* and weaker activity towards *F. poae* and *F. graminearum*.

It is known from the literature that citronellol showed a weak or not detectable activity against several species of toxigenic fungi, such as *Aspergillus*, *Penicillium* and *Eurotium* [21]. On the contrary, when the researchers have investigated the inhibitory effects of citronellol against strains of *Trichophyton rubrum*, this compound showed a good antifungal activity [22]. In another work citronellol showed a weak activity against *F. oxysporum* f. sp. *gladioli*, when compared with carvacrol and thymol antifungal activity [23]. In the literature there are no data concerning the antifungal activity of citronellol against other *Fusarium* species and strains of *Alternaria* and *Phytophtora.* It can therefore be assumed that the activity carried out by VEO could be entirely due to the contribution of its main components, citral and citronellol, since more than 70% of the composition of VEO is made up of these compounds. The antifungal activity is considerably influenced by citral due to its well documented effects on fungi.

## 3. Materials and Methods

### 3.1. Plant Material and Essential Oil

VEO was obtained by steam distillation from leaves of *V. macrophylla* which were collected in the eastern coastal forest of Madagascar (Sahamamy/Analalava, district of Mahavelona Toamasina II) in May 2011. The essential oil was chemically analyzed by GC-FID and GC-MS according to Maggi et al. [16,20].

### 3.2. Fungi Strains

The VEO was tested against six phytopathogenic fungi from the collection of Botanic Garden of Urbino University—*Fusarium* strains: *F. avenaceum*, *F. poae*, *F. graminearum*, *F. semitectum*; *Alternaria* strain: *A. solani*; *Phytophthora* strain: *P. cryptogea.* The phytopathogenic fungi were cultured in appropriate culture media (for *Fusarium* species and *A. solani* the culture medium was PDA potato dextrose agar; for *P. cryptogea* the culture medium was V8-Juice agar). In vitro antifungal activity of VEO against phytopathogenic fungi was carried out according to the agar dilution method [24,25].

### 3.3. Agar Dilution Method

Briefly, potato dextrose agar (PDA) and V8-Juice agar plates were prepared using 9 cm diameter Petri dishes. VEO was dissolved in absolute ethyl alcohol and 5% Tween 20 (Fluka) was added in order to obtain an emulsion. Aliquots of this emulsion were added to the culture medium at a temperature of 40–45 °C and then poured into Petri dishes (Ø 9 cm) [24,25]. Concentrations of 100, 200, 400 and 800 µg/mL of VEO were tested. A disc (5 mm diameter) of the fungal species was cut from 1-week-old cultures PDA plates (for *Fusarium* species and *A. solani*) and V8-Juices agar medium (for *P. cryptogea*). Then the mycelia surface of the disc was placed upside down on the center of a Petri dish with the culture medium containing different concentrations of essential oil. Then, the plates were incubated in the dark at 22 ± 2 °C. Controls consisted of 100, 200, 400 and 800 µg/mL of the emulsion described above, where VEO was replaced with sterile distilled water. In addition, Nystatin was used as reference fungicide. The treatments were incubated under controlled temperature conditions of 22 ± 1 °C in the dark. The diameters of the fungal growth were measured after 1, 3 and 6 days. The fungal growth in the control treatments was considered when it had completely covered the Petri dishes. The percentage of growth inhibition by treatment was calculated from the following equation: Mycelial growth inhibition (%) = (DC−DT)/DC × 100, where DC and DT are average diameter of fungal colony of control and treatment, respectively.

The fungicidal activity of the oil was determined using the technique of Carta and Arras and Thompson [26,27]. The mycelia disks were transferred from Petri dishes in which no growth was observed (total inhibition = 100) onto fresh plates of culture medium in order to verify after 7 days the fungistatic or fungicidal activity of such inhibition. For evaluation of the fungistatic/fungicidal activity the reading was done visually. VEO was considered fungicidal when no mycelial growth in the Petri dishes containing medium culture was observed. All experiments were carried out in triplicate.

### 3.4. Determination of Minimum Inhibitory Concentration (MIC)

The MIC values (µg/mL) were determined by the dilution method in solid medium. Dilutions of the emulsions of oil were made in the culture medium over the concentration range of 100 to 200 µg/mL for *P. cryptogea* and *F. avenaceum*, of 200 to 400 µg/mL for *F. poae* and *F. graminearum*, of 50 to 100 µg/mL for citral and of 400 to 800 µg/mL for citronellol. MICs were determined as the concentration with no visible growth. All experiments were performed in triplicate, if the MIC results were different only higher value obtained was noted.

### 3.5. Determination of Minimum Fungicidal Concentration (MFC)

In order to determine the fungicidal activity (MFC) of VEO a membrane filtration method was used [28]. After the reading of the MICs, about 10µl of each sample, were transferred into PDA plates for *Fusarium* species and int V8-Juice agar for *P. cryptogea*.

After 24 h of incubation at 35 °C the reading of the minimum fungicidal concentration as the lowest concentration in which there was no growth of the fungus was revealed. MFCs values were calculated from 100 to 200 µg/mL for *P. cryptogea* and *F. avenaceum*. For *F. poae* and *F.graminearum* MFCs were calculated from 200 to 400 µg/mL. All experiments were performed in triplicate.

### 3.6. Statistical Analysis

Analysis of variance was performed by One-way ANOVA and by Duncan’s post hoc test. Statistical differences at *p* < 0.05 were considered to be significant.

## 4. Conclusions

EOs own two prominent features, i.e., (i) low toxicity for people and the environment due to their natural properties and (ii) low risk for resistance development by pathogenic microorganisms.

The current investigation highlighted the antifungal potential of VEO against *F. avenaceum* and *P. cryptogea* and a fungistatic activity against all the other fungi.

It is important to underline that many researchers have shown that the inhibitory effects of EOs vary according to some different parameters such as the type of fungus considered, the different indicateresponse mechanism in relation to the variability of the EO components, their structural configuration and possible synergistic actions.

Since this work showed that not all fungi responded in the same way to the presence of VEO, it can be assumed that the same result can be obtained in other screenings with other genera of fungi. Therefore, it would be interesting to carry out a further study considering different species that cause other important diseases in order to use a wide range of results for the promotion and development of new selective and natural fungicides from this Malagasy endemic plant.

## Figures and Tables

**Table 1:** Effect of *Vepris macrophylla* leaf essential oil (VEO) on the in vitro growth of selected fungal pathogens (% inhibition).

		Positive Control	Concentration (µg/mL)			
Fungi	Days	50 (µg/mL)	100	200	400	800
*A. solani*	1°	57.5 ± 6.99 aA	20.1 ± 5.30 bD	25.9 ± 6.48 bG	24.3 ± 3.15 bI	45.8 ± 4.56 cN
	3°	78.0 ± 2.20 dB	30.4 ± 3.95 eE	30.6 ± 3.75 eG	34.2 ± 4.50 eL	53.8 ± 2.75 fN
	6°	100.0 ± 0.00 gC	57.2 ± 6.98 hF	60.8 ± 7.88 hH	66.3 ± 4.90 hM	73.3 ± 7.00 iO
*P. cryptogea*	1°	57.5 ± 6.99 aA	23.7 ± 3.80 bD	55.3 ± 4.56 aF	60.0 ± 2.41 aH	100.0 ± 0.00 cM
	3°	78.0 ± 2.20 dB	31.5 ± 3.31 eD	62.6 ± 3.01 fF	77.0 ± 2.05 dI	100.0 ± 0.00 gM
	6°	100.0 ± 0.00 hC	73.8 ± 5.51 iE	100 ± 0.00 hG	100.0 ± 0.00 hL	100.0 ± 0.00 hM
*F. avenaceum*	1°	60.0± 6.99 aA	23.5 ± 3.31 bD	36.1 ± 3.97 cG	60.0 ± 2.40 aL	72.5 ± 1.88 dO
	3°	88.0 ± 7.20 eB	34.2 ± 5.92 fE	62.8 ± 3.59 gH	77.7 ± 2.47 hM	82.3 ± 3.60 eO
	6°	100.0 ± 0.00 iC	74.9 ± 7.14 lF	100.0 ± 0.00 iI	100.0 ± 0.00 iN	100.0 ± 0.00 iP
*F. poae*	1°	67.5 ± 6.99 aA	35.9 ± 3.72 bC	37.2 ± 4.13 bE	39.4 ± 1.43 bG	42.2 ± 4.5 bL
	3°	75.0 ± 5.20 cA	43.5 ± 4.12 dC	47.0 ± 3.60 dE	52.1 ± 4.45 dH	60.1 ± 7.06 eM
	6°	100.0 ± 0.00 fB	66.3 ± 4.95 gD	72.8 ± 5.26 gF	100.0 ± 0.00 fI	100.0 ± 0.00 fN
*F. graminearum*	1°	57.5 ± 6.99 aA	45.1 ± 3.82 bD	53.3 ± 2.08 aF	62.8 ± 2.65 aI	62.8 ± 2.65 aN
	3°	77.0 ± 5.20 cB	51.0 ± 1.40 dD	70.0 ± 5.02 cG	71.0 ± 3.81 cL	71.0 ± 3.81 cN
	6°	100.0 ± 0.00 eC	70.0 ± 7.17 fE	88.0 ± 8.51 gH	100.0 ± 0.00 eM	100.0 ± 0.00 eO
*F. semitectum*	1°	57.5 ± 6.99 aA	39.4 ± 3.78 bD	54.4 ± 5.41 aF	61.4 ± 1.40 aH	67.1 ± 3.65 aL
	3°	79.0 ± 6.20 cB	45.7 ± 2.23 dD	59.4 ± 4.96 eF	62.5 ± 3.59 eH	67.4 ± 3.14 eL
	6°	100.0 ± 0.00 fC	59.1 ± 5.70 gE	70.1 ± 4.13 hG	74.7 ± 4.16 hI	78.7 ± 5.67 hM

The values are the average of three determinations. Positive control was represented by nystatin; Different lowercase letters within a row indicate significant differences between means (*p* < 0.05); Different uppercase letters within a column referring to a fungal species, indicate significant differences between means (*p* < 0.05).

**Table 2:** Minimal inhibitory concentration (MIC) and minimal fungicidal concentration (MFC) of *Vepris macrophylla* essential oil, citral and citronellol.

Fungi	Essential Oil		Citral		Citronellol		Nystatin	
	MIC (µg/mL)	MFC (µg/mL)	MIC (µg/mL)	MFC (µg/mL)	MIC (µg/mL)	MFC (µg/mL)	MIC (µg/mL)	MFC (µg/mL)
*P. cryptogea*	130.40a	390.30d	100e	510f	510g	790h	45i	50m
*F. avenaceum*	130.40a	390.30d	100e	510f	510g	790h	45i	50m
*F. poae*	300.20b	/*	100e	510f	510	>800	45i	50m
*F. graminearum*	215.00c	/*	100e	510f	510g	>800	45i	50m

The values are the average of three determinations; Positive control represented from nystatin showed MIC and MFC values. corresponding to 45 µg/mL and 50 µg/mL. Different letters within a column indicate significant differences between the mean (*p* < 0.05) * For *F. poae* and *F. graminearum* at concentration >800 µg/mL no fungicidal activity was detected, but also a fungistatic activity was revealed.

## Abbreviations

VEO	*V. macrophylla* leaf essential oil
EOs	Essential oils
*F. avenaceum*	*Fusarium avenaceum*
*F. poae*	*Fusarium poae*
*F. graminearum*	*Fusarium graminearum*
*F. semitectum*	*Fusarium semitectum*
*A. solani*	*Alternaria solani*
*P. cryptogea*	*Phytophthora cryptogea*
MIC	Minimal Inhibitory Concentration
MFC	Minimum Fungicide Concentration
